# Risk Factors of In-Hospital Venous Thromboembolism and Prognosis After Emergent Ventral Hernia Repair

**DOI:** 10.1155/2024/6670898

**Published:** 2024-11-12

**Authors:** Wei Yang, Jie Ling, Yun Zhou, Pengcheng Yang, Jiejing Chen

**Affiliations:** ^1^Department of General Surgery, The Affiliated Hospital of Yangzhou University, Yangzhou 225000, Jiangsu, China; ^2^Department of Vascular Surgery, The Affiliated Hospital of Yangzhou University, Yangzhou 225000, Jiangsu, China; ^3^Department of Pediatrics, The Affiliated Hospital of Yangzhou University, Yangzhou 225000, Jiangsu, China

**Keywords:** emergent ventral hernia repair, mortality, nomogram, prognosis, venous thromboembolism

## Abstract

**Background:** The risk factors and association of venous thromboembolism (VTE) following emergent ventral hernia repair (EVHR) remains uncertain. This aim of the study aims was to establish the predictors of VTE after EVHR and its influence on the long-term outcomes.

**Methods:** A total of 2093 patients from the MIMIC-IV database who underwent EVHR were recruited. Multivariate logistic regression and nomogram models were developed to predict in-hospital VTE and mortality. Calibration and receiver operating characteristic (ROC) curves were utilized to assess the model's effectiveness and reliability. Decision curve analysis (DCA) was performed to evaluate the net clinical benefits of the model.

**Results:** The rate of in-hospital VTE was 1.6% (33/2093) after EVHR. Four independent potential factors were established after multivariate analysis, and the abovementioned risk factors fit into the nomogram. The prediction model presented good performance metrics (C-index: 0.857), the calibration and ROC curves demonstrated the accurate prediction power, and DCA indicated the superior net benefit of the established model. In-hospital and 1-year mortality rates were 0.8% (17/2093) and 4.1% (86/2076) after EVHR. The potential factors were included in the mortality prediction nomogram. The prediction model presented good performance metrics (C-index of 0.957 and 0.828, respectively), the calibration and ROC curves were consistent with the actual results, and DCA indicated the superior net benefit of the established model.

**Conclusion:** The nomogram, derived from the logistic regression model, demonstrated excellent predictive performance for VTE occurrence and prognosis in patients following EVHR. This model could serve as a valuable reference for clinical decision-making regarding VTE prevention and for enhancing post-EVHR prognosis.

## 1. Introduction

Venous thromboembolism (VTE) refers to abnormal blood coagulation in veins, leading to complete or partial obstruction of blood vessels. VTE ranks as the third most common cardiovascular disease following ischemic heart disease and stroke [1], encompassing conditions such as deep vein thrombosis (DVT), pulmonary embolism (PE), and splanchnic vein thrombosis (SVT). Moreover, VTE represents a potentially serious complication of surgeries, significantly impacting patient's prognosis and quality of life. Particularly in emergency general surgery (EGS) patients, VTE incidence is notably high, with approximately 2.5% experiencing an in-hospital VTE event [2]. About 34% of in-hospital mortality attributed to by PE, with VTE representing the most preventable cause of morbidity and mortality in hospitalized patients [3]. Accurate and timely prediction is paramount to preventing VTE [4], and numerous VTE risk-assessment tools are presently available. In addition, guidelines advocate for appropriate VTE prophylaxis as a key patient safety practice [5]. The prevention of VTE has garnered increased attention from emergency general surgeons in recent years.

Ventral hernia is a prevalent condition in general surgery, with approximately 10% of ventral hernia patients necessitating emergent surgery [6]. Ventral hernias encompass groin (inguinal and femoral) and anterior ventral (umbilical, parastomal, and incisional) hernias. Emergent ventral hernia repair (EVHR) is linked to increased postoperative complications and poorer prognosis compared with elective ventral hernia repair [7]. Several observational studies have indicated that patients undergoing EGS face a heightened risk of VTE, and accurate assessment of VTE risk coupled with optimal prophylaxis significantly enhances clinical practices [8]. Nonetheless, there is a paucity of research on the mortality and prognosis of EVHR patients. Furthermore, the incidence of VTE in EVHR patients remains unknown, despite some studies examining VTE incidence in inguinal hernia.

The incidence of ventral hernia among the elderly rises, often accompanied by multiple underlying diseases. Some patients opt for a watchful waiting approach, weighing the challenges associated with surgical intervention. Given the elevated risk of complications following EVHR, timely treatment becomes imperative, particularly for patients with compromised general health. The Caprini risk score has proven effective in predicting postoperative VTE [9]. In this study, we investigated additional risk factors of postoperative VTE and assess in-hospital mortality and prognosis following EVHR. Furthermore, we developed a prediction model specialized for VTE risk and mortality, offering a clinically valuable tool for predicting in-hospital VTE and mortality based on the Medical Information Mart for Intensive Care IV (MIMC-IV) database.

## 2. Methods

The MIMIC-IV database contains high-quality data on patients admitted to emergency and intensive care units (ICUs) at the Beth Israel Deaconess Medical Center between 2008 and 2019 (https://mimic.mit.edu/). MIMIC-IV is a large longitudinal database with 1-year follow-up data. The present study was approved to obtain access to the database and was responsible for data extraction (51,946,791).

### 2.1. Source of Data and Research Subjects

Relevant data from 2093 patients were retrieved from the MIMIC-IV database spanning from 2008 to 2019. The primary diagnosis, clinical characteristics, laboratory examinations, and procedures were obtained using PostgreSQL and Navicat based on distinct ICD-9 codes. We selected all patients who underwent EVHR, and those diagnosed with VTE(+) patients had either DVT, PE, or SVT, with VTE diagnosis confirmed through various imaging examinations. Exclusion criteria included (1) censored data exceeding 40% and (2) incomplete variables.

### 2.2. Data Extraction

We extracted data from the MIMIC-IV database using PostgreSQL and Navicat Premium. The variables included demographic information, comorbidities, treatments, procedures, postoperative complications, laboratory parameters, and observation endpoints, including in-hospital and 1-year mortality. Demographic information encompassed age, gender, BMI, race, history of smoking, history of VTE, history of anticoagulation, and comorbidity data (Charlson Comorbidity Index [CCI] collected for analysis based on ICD-9 codes). The CCI served as the model used to predict mortality for patients with a range of comorbid conditions [10]. Procedure-associated treatments during hospitalization included the hernia repair type, laparoscopic operation, bowel resection (enterotomy), and mesh application. The treatment process included length of hospitalization (LOH), interval from admission to surgery, postoperative anticoagulation, and postoperative complications such as bleeding, infection, fistula, and abnormal reactions caused by the implant. Laboratory variables measured upon admission included partial indicators of complete blood count, white blood cell (WBC) count, hematocrit, platelet count (PLT) , and partial biochemical indicators, such as anion gap, bicarbonate, blood urea nitrogen (BUN) calcium, chloride, creatinine, glucose, sodium, and potassium levels.

### 2.3. Nomogram Construction and Statistical Analysis

Univariate and multivariate logistic regression analyses were employed to assess the independent risk factors influencing in-hospital VTE and mortality in patients after EVHR, while Cox regression analyses were utilized to evaluate the independent risk factors influencing 1-year mortality in these patients. The variables included in the multivariate logistic regression analysis were selected based on the results of the univariate regression analysis (*p* < 0.1). A univariate *p* value < 0.05 was considered necessary for a variable to be included in the final prediction model. The effect measure of each variable is presented as odds ratios (ORs) or hazard ratios (HRs) with the corresponding 95% confidence intervals (CIs). The interactive nomogram was constructed using the “RMS” and “regplot” R packages in Rstudio.

The model's predictive performance was assessed through internal validation using the bootstrap method. Performance was further quantified in terms of calibration with the calibration curve incorporating the Hosmer–Lemeshow goodness-of-fit test. A *p* value in the Hosmer–Lemeshow test > 0.05 indicates that the calibration model was satisfactory. The Model discrimination was evaluated using the consistency index (C-index) and receiver operating characteristic (ROC) curve analysis. In addition, the area under the ROC curve (AUC) was calculated to quantitatively assess the model's discriminative ability. Finally, decision curve analysis (DCA) was conducted to determine the clinical utility of the model in guiding clinical decision-making.

SPSS 26.0 (IBM, USA) and Rstudio (https://rstudio.com) were utilized for statistical analyses. Measurement data with a normal distribution were expressed as means (*x*) ± standard deviations (*s*), and comparisons between two groups were conducted using a *t*-test. Categorical data were presented as frequencies and compared between groups by the *χ*^2^ test. The continuous age variable was categorized based on commonly used clinical practice cutoff values. Logistic regression was employed to analyze the risk factors for VTE and mortality, while Cox regression was utilized to analyze prognostic risk variables. The efficacy and goodness of fit were assessed using ROC and calibration curves. Survival analysis was conducted using log-rank analysis and Kaplan–Meier (KM) curves. A *p* < 0.05 was considered statistically significant.

## 3. Results

### 3.1. Patient Characteristics and VTE Incidence

According to the inclusion and exclusion criteria, 2093 patients were included from the MIMIC-IV database. The in-hospital VTE rate was 1.6%.  Table 1 presents the demographic and clinical information of all 2093 patients. Among them, 52.8% were male and 34.7% were over 65 years old, with a mean BMI of 31.2 ± 8.0. In addition, 72.3% were White, 10.5% had a history of smoking, 5% had a history of VTE, 8.2% had a history of anticoagulation, and 10.1% were transferred to the ICU after EVHR. Moreover, over 78.4% of the patients had an anterior ventral hernia (umbilical, parastomal, and incisional), while 21.6% had a groin hernia (inguinal and femoral). Laparoscopic operations were performed in 10.9% of the patients, with all VTE patients undergoing open surgery. In addition, 6.5% of the patients underwent intestinal resection and 71.9% received a hernia mesh. Subcutaneous administration of 5000 units of unfractionated heparin (UFH) after surgery was performed in 84.9% of the patients, with postoperative bleeding occurring in 0.7%. No significant difference was detected in bleeding incidence among patients with postoperative prophylactic anticoagulation (OR = 0.65, 95% CI: 0.18–2.35, and *p*=0.51). Furthermore, 3.2% of the patients developed a postoperative infection, while 0.9% experienced persistent postoperative fistula, a serious complication. Notably, over 70% of the patients received a surgical mesh, with 3.3% developing mesh-related complications. Significant differences were identified in hematocrit and chloride levels; however, other laboratory parameters did not show significant differences.

### 3.2. Logistic Regression Analysis of Risk Factors for VTE

Initially, 31 potential predictors were included in the univariate regression analysis, revealing that race, CCI, ICU stay, LOH, hernia type, postoperative anticoagulation, interval from admission to surgery, intestinal resection, mesh application, postoperative infection, fistula, hematocrit, and chloride levels were statistically associated with the risk of in-hospital VTE after EVHR (*p* < 0.05). Additionally, three variables with *p* < 0.1 (history of VTE, abnormal reaction caused by the implant, and bicarbonate levels) were also included in the multivariate regression analysis. Consequently, 16 candidate predictive factors were incorporated into the multivariate model, with four variables (postoperative fistula, intestinal resection, LOH and postoperative anticoagulation) were ultimately identified as predictors of in-hospital VTE after EVHR (Table 2).

### 3.3. In-Hospital and 1-Year Mortality Rates After EVHR

The in-hospital and 1-year mortality rates were 0.8% (17/2093) and 4.1% (86/2076), respectively, after EVHR. The most common causes of in-hospital and 1-year mortality included acute mesenteric ischemia, intestinal perforation, severe sepsis, septic shock, acidosis, acute kidney failure, acute respiratory failure, acute heart failure, and postoperative complications such as peritoneal adhesions and intra-abdominal infection (Table 3).

### 3.4. Univariate and Multivariate Analysis of Factors Influencing In-Hospital and 1-Year Mortality

**Figure 1:**
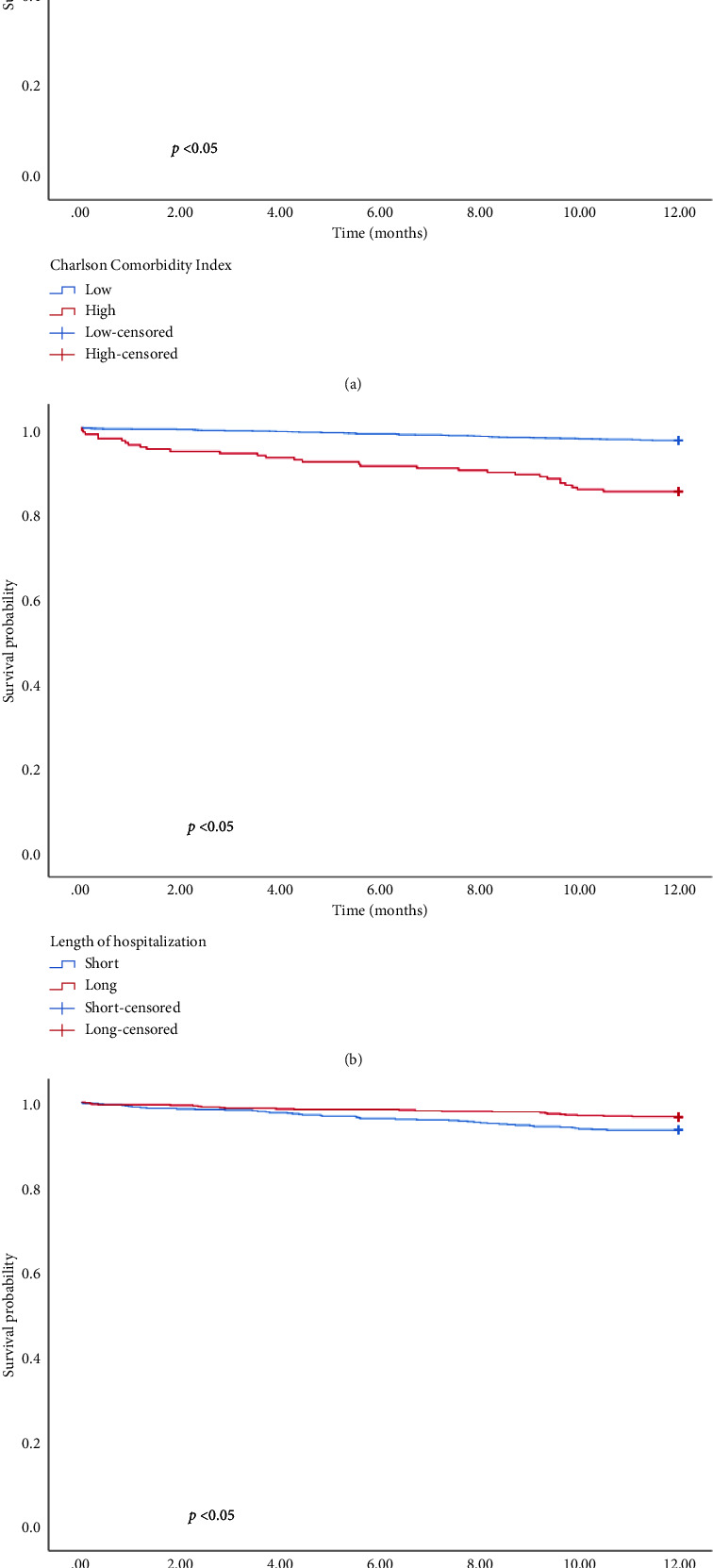
KaplanMeier curves in prediction of 1-year survival for (a) Charlson Comorbidity Index, (b) length of hospitalization, and (c) hematocrit in patients after EVHR.

The univariate and multivariate analysis models are presented in Tables 4 and 5, respectively. Significant differences were observed in the CCI, smoking history, and intestinal resection for in-hospital mortality and CCI, LOH, and hematocrit for 1-year mortality. Patients with a higher CCI had an elevated risk of in-hospital (OR = 1.646, 95% CI: 1.308–2.072, and *p* < 0.01) and 1-year mortality (OR = 1.325, 95% CI: 1.199–1.464, and *p* < 0.01). The risk of in-hospital mortality for patients with a smoking history was 5.324 times higher than for those without (OR = 5.324, 95% CI: 1.260–22.502, and *p*=0.02). Similarly, patients undergoing intestinal resection had an 11.597 times higher risk of in-hospital mortality compared to those without (OR = 11.597, 95% CI: 3.288–40.909, and *p* < 0.01). Furthermore, 1-year mortality was higher in patients with longer hospitalization durations compared with those with shorter stays (OR = 1.027, 95% CI: 1.002–1.053, and *p*=0.04). Patients with higher hematocrit levels exhibited a better prognosis than those with lower levels (HR = 0.927, 95% CI: 0.877–0.980, and *p* < 0.01). In addition, hematocrit, LOH, and CCI were used to categorize patients into low and high groups. The KM survival analysis demonstrated that CCI, LOH, and hematocrit were associated with 1-year survival (Figure 1).

### 3.5. Construction of the VTE Prediction Nomogram Model

**Figure 2:**
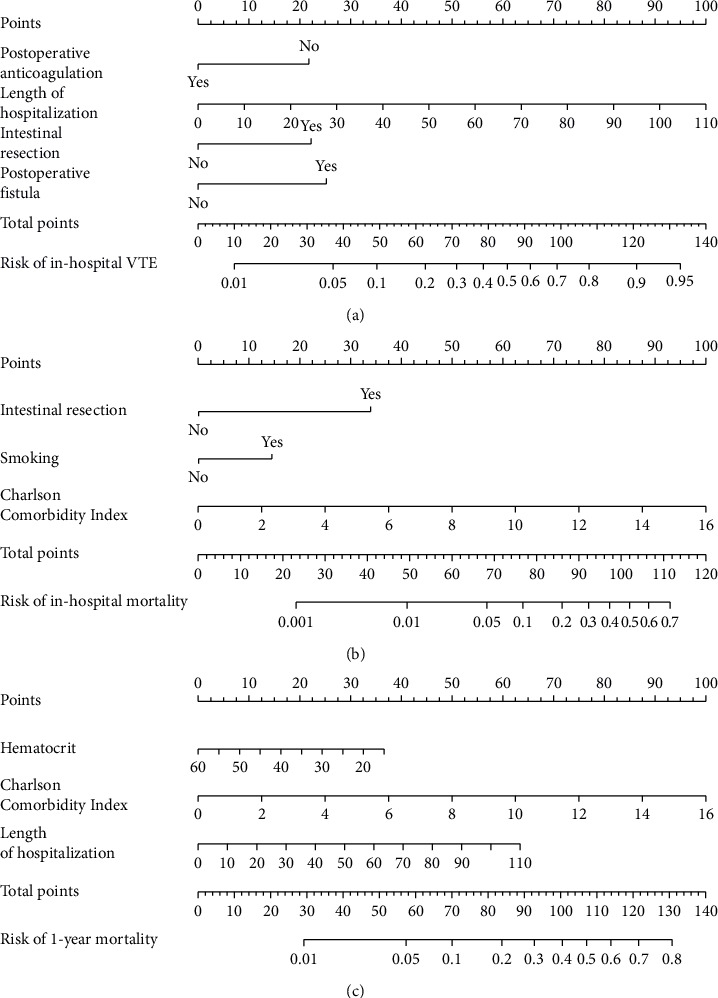
The nomogram models for prediction of (a) in-hospital VTE, (b) in-hospital mortality, and (c) 1-year mortality in patients after EVHR.

Based on the multivariate logistic regression analysis described above, we developed a predictive model for in-hospital VTE incorporating the significant variables: postoperative fistula, intestinal resection, LOH, and postoperative anticoagulation. The prediction model demonstrated good performance metrics with a C-index of 0.857. To facilitate clinical application, we constructed a nomogram containing these four variables for model visualization. The likelihood of VTE is directly proportional to the total score obtained from each variable, allowing for convenient clinical use.  Figure 2(a) illustrates the scores assigned to each variable in the nomogram, enabling direct determination of the probability of developing VTE.

### 3.6. Construction of In-Hospital and 1-Year Mortality Prediction Nomogram Models

We established an in-hospital mortality prediction nomogram using variables identified from multivariate logistic regression analysis and 1-year mortality prediction nomogram using variables identified from Cox regression analysis. The in-hospital mortality model included significant variables (CCI score, smoking history, and intestinal resection), while the 1-year mortality model included significant variables (LOH, CCI score, and hematocrit). Both prediction models demonstrated good performance metrics with a C-index of 0.957 and 0.828, respectively. Each variable was projected onto the scale to directly obtain the total score reflecting the in-hospital and 1-year mortality risk (Figures 2(b) and 2(c)).

### 3.7. Model Calibration and Discrimination Performance

**Figure 3:**
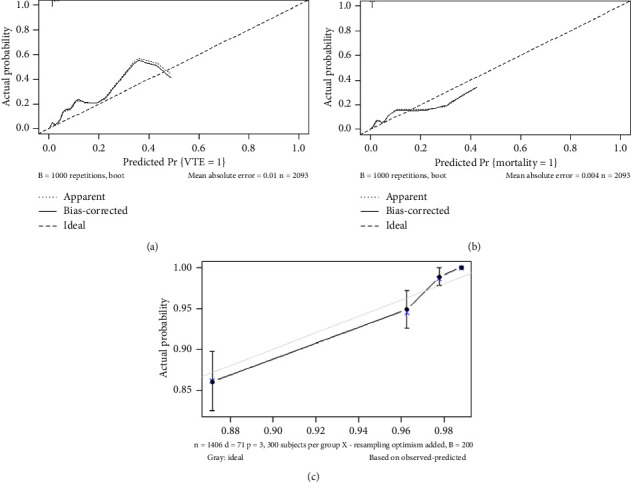
Calibration plots of the nomogram in the prediction of (a) in-hospital VTE, (b) in-hospital mortality, and (c) 1-year mortality in patients after EVHR.

**Figure 4:**
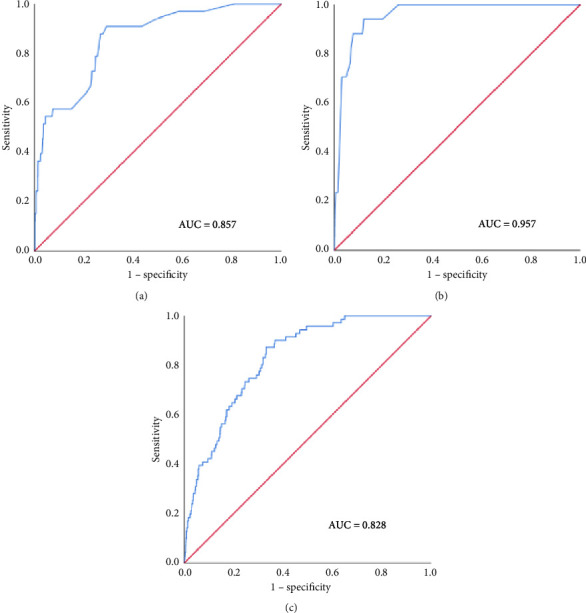
ROC curves for (a) in-hospital VTE, (b) in-hospital mortality, and (c) 1-year mortality in patients after EVHR.

The internal calibration curves, drawn using the bootstrap method, closely matched the standard curve (Figure 3), indicating that the nomogram prediction model demonstrated good predictive value with internal data. Model discrimination was assessed by measuring the AUCs. The AUC for the VTE prediction model was 0.857 (95% CI: 0.796–0.918), for the in-hospital mortality model, it was 0.957 (95% CI: 0.930–0.984), and for the 1-year mortality model, it was 0.828 (95% CI: 0.788–0.868). These results suggest that all prediction models exhibited good discrimination (Figure 4).

### 3.8. Clinical Utility of the Model

**Figure 5:**
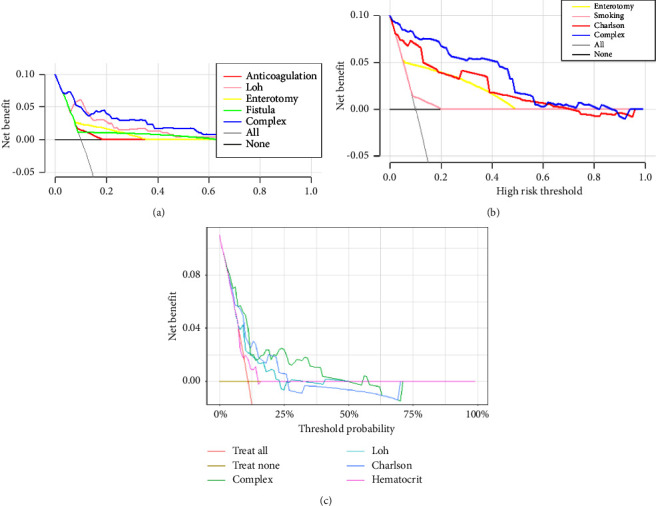
Decision curve analysis (DCA) for assessment of the clinical utility for nomogram in the prediction of (a) in-hospital VTE, (b) in-hospital mortality, and (c) 1-year mortality in patients after EVHR.

The decision curves for the predictive nomograms are presented in Figure 5. Within a risk threshold range of 0.20–0.60, the complex nomogram models demonstrated higher net profits compared to models considering all, single, or no variables.

## 4. Discussion

Our study investigated the rate of in-hospital VTE after EVHR, identifying four independent potential factors through multivariate analysis. In addition, we examined the in-hospital and 1-year mortality rates after EVHR. While models predicting postoperative VTE exist, research specifically focusing on patients with EVHR is lacking. In our study, we identified 16 clinical features from more than 30 that may relate to postoperative VTE in patients after EVHR and constructed a VTE prediction model. Surgery constitutes the primary treatment for ventral hernia, encompassing various surgical types and potential postoperative complications. Following EVHR, patients face heightened risks of VTE due to postoperative trauma and immobility, among other factors.

Currently, numerous consensus statements and guidelines exist for preventing and treating postoperative VTE. For instance, European guidelines for perioperative VTE prevention recommend identifying pre-existing comorbidities that may increase the risk of postoperative VTE and using low-molecular-weight heparin for postsurgery patients [11]. Previous reports suggest the incidence of postoperative VTE after general surgery ranges from 10% to 40% [12], while the incidence of perioperative VTE in inguinal hernia cases ranges from 0.18% to 0.45% [13]. However, the quality of these studies varies [14]. Therefore, this study aims to investigate the incidence of perioperative VTE after EVHR in a large dataset.

Several factors contributing to postoperative VTE have been identified following hernia surgery. A study involving 11,707 Chinese patients after hernia surgery explored five variables related to in-hospital VTE: varicose veins of the lower extremity, VTE history, thrombosis, anticoagulation, and reducible hernia [15]. The CHAT-1 study, which utilized machine learning to predict in-hospital VTE after inguinal hernia repair, investigated 15 combined variables as VTE predictors [16]. Another study on postoperative VTE after incisional ventral hernia repair indicated that component separation, duration of operation, and Body Mass Index (BMI) are significant factors contributing to postoperative VTE, accounting for 7.9% of the total cohort [17].

However, few studies have constructed nomograms to predict VTE, especially after EVHR. Hence, we focused on VTE and mortality risks after EVHR and found that postoperative prophylactic anticoagulation was inversely associated with VTE. Herein, 84.9% of the patients received different frequencies of UFH subcutaneous administration after surgery, with 31% receiving 5000 u three times a day, 25.6% receiving 5000 u twice a day, and 28% receiving 5000 u once a day. We also observed a higher rate of early postoperative prophylactic anticoagulation in non-VTE patients compared with VTE patients, suggesting that early postoperative prophylactic anticoagulation could reduce the incidence of VTE. Although 69% of VTE patients underwent postoperative prophylactic anticoagulation with UFH, VTE still occurred in these patients. Previous studies have shown that the surgical type, VTE, bleeding risk factors, and drug interactions should be considered to allow for an appropriate and personalized approach for thromboprophylaxis [18], and low-molecular-weight heparin is more effective than UFH for the prevention of postoperative VTE [19]. Coagulation function monitoring was recommended for all patients undergoing operations routinely. We speculate that patients at high risk of VTE may benefit from stronger anticoagulant treatments compared to prophylactic anticoagulation with UFH. Therefore, the appropriate dosage and choice of anticoagulants might be associated with the efficacy of thromboprophylaxis.

Previous studies have indicated that the length of hospital stay influences VTE prophylaxis patterns and events. VTE events tend to increase with longer hospitalizations for patients with acute illnesses [20]. In addition, the prevalence of VTE after total joint arthroplasty is associated with the length of stay [21]. In our study, we found that VTE was associated with the length of stay of patients after EVHR. Morbidity and mortality rates were higher in urgent ventral hernia cases compared with elective surgeries. The presence of intestinal resection and hernia type increased morbidity [22], which is consistent with our current results. Specifically, 135 patients in our study underwent intestinal resection, and VTE was significantly associated with both intestinal resection and postoperative fistula. A high prevalence of VTE has been reported after abdominal surgeries, including intestinal resection [23]. VTE might also be associated with patient positioning during surgery and comorbidities [24]. The increased risk of bleeding limited VTE prophylaxis, especially in patients undergoing intestinal resection. Randomized controlled trials (RCTs) have demonstrated that patients undergoing abdominal surgery and receiving fondaparinux had low bleeding incidence [19]. Another noninferiority RCT showed that fondaparinux could reduce VTE risk with no significantly different bleeding rates [25]. These studies support the safety and efficacy of VTE prophylaxis. Nevertheless, the risk of bleeding events should still be taken seriously, and constructing a bleeding risk assessment model would also be clinically useful.

Furthermore, we incorporated other surgery-associated risks and obtained laboratory examinations. A previous study found that the risk of VTE was 2.6-fold higher for patients with infection after revision total knee replacement [26]. Another multicenter study suggested that postoperative complications, such as anastomotic leak or fistula, abdominal abscess, and infection were risk factors for VTE after bariatric surgery [27]. Our present results indicated that patients with postoperative infection had a higher risk of VTE. Mesh application is not commonly used to reduce the risk of infection for incarcerated or strangulated hernias requiring enterectomy. The VTE incidence was significantly lower in laparoscopic surgery than in open surgery [28]. This is consistent with the current results, showing that open surgery led to a higher incidence of VTE than laparoscopic surgery. Plasma D-dimer concentration is considered an important variable in predicting VTE occurrence. However, coagulation markers were not included in the present study because the censored data exceeded 40%. Although a significant difference was detected for chloride levels in the univariate analysis, it did not persist in the multivariate analysis. Previous studies have shown that mortality increased among patients with VTE after hepatectomy [29]. Similarly, VTE after nephrectomy was associated with higher mortality rates [30]. Nevertheless, our results showed that VTE was not associated with in-hospital and 1-year mortality due to the low incidence of VTE and accurate treatment.

There have been few studies focusing on in-hospital mortality previously, and our study also investigated the 1-year prognosis of patients after EVHR. The in-hospital and 1-year mortality rates were 0.8% (17/2093) and 4.1% (86/2076), respectively, in this study, indicating that emergency surgery was associated with high postoperative morbidity and mortality. Previous studies have shown that increasing age and intestinal resection were associated with mortality after incisional hernia repair [31]. Similarly, a nationwide cohort study including 9741 patients undergoing emergency groin hernia repair demonstrated that mortality was influenced by increasing age, comorbidity, and intestinal resection [32]. Both univariate and multivariate analyses indicated that the CCI was associated with complications and mortality after emergency hernia repair in the elderly [33]. Independent predictors of emergency repair included CCI, which was associated with a greater odd of in-hospital death [34]. Consistently, our study also demonstrated that the CCI was associated with both in-hospital and 1-year mortality. Therefore, ventral hernia patients with high comorbidity should receive increased attention to prevent high mortality after EVHR.

Smoking was identified as an independent predictor of 30-day mortality in patients undergoing EGS [35]. Our study found that smoking was associated with in-hospital mortality, indicating that smoking patients were at a higher risk of death than nonsmoking patients. Smoking has many potential effects on general condition, including inflammation, leukocyte adhesion, and platelet activation [36]. Intestinal resection resulted in a considerably poorer postoperative outcome with a mortality rate of 7.5%. Explorative laparoscopy assessed the hernia sac contents and blood flow to the strangulated bowel segment, while patients undergoing intestinal resection faced a poorer prognosis and higher mortality [37]. Patients in the ICU would be accompanied by more comorbidities and complications, and more accurate outcome prediction models, including ICU and hospital stay, would be useful to guide clinical decision-making [38]. Here, we showed that admission to the ICU was significantly associated with in-hospital mortality, and increased LOH was associated with 1-year mortality.

Few studies have evaluated prognostic factors for EVHR, making the identification of critical prognostic biomarkers essential for assessing patients' conditions and guiding subsequent treatments. A previous study established that routine blood parameters, including hematocrit, were associated with disease severity in congenital diaphragmatic hernias [39]. In addition, hematocrit levels were identified as an independent risk factor for increased 30-day mortality in sepsis patients [40]. Hematocrit levels might serve as prognostic indicators for various diseases and in the general population [41]. Here, we demonstrate for the first time that hematocrit is associated with the 1-year prognosis after EVHR, possibly due to decreased red blood cell infusion affecting oxygen transport and metabolism.

The MIMIC-IV database represents the first large ICU database available to the public, encompassing various diseases. Our study marks the first investigation into EVHR based on the MIMIC-IV database. Nonetheless, there are several limitations to consider. First, this study is retrospective, leading to potential selection bias, and lacks specific information on preoperative anticoagulation and postoperative nonpharmacological VTE prevention. Second, the study primarily involves White subjects, with few non-White individuals included, potentially impacting the generalizability of the results due to racial differences. Third, certain important surgery-associated risk factors and laboratory examinations, such as the surgery duration and type of anesthesia, were not available in this database. Lastly, the low incidence of VTE after EVHR weakens the reliability of the predictive model, and external validation was not conducted. Future research should consider multicenter studies with sufficient VTE cases after EVHR to validate this prediction model effectively.

## 5. Conclusions

In summary, clinical parameters, laboratory examinations, and therapeutic factors were integrated into a nomogram model to predict VTE and mortality after EVHR. Postoperative fistula, intestinal resection, LOH, and postoperative anticoagulation were finally identified as predictors of in-hospital VTE. CCI, smoking, and intestinal resection were used to predict in-hospital mortality. The 1-year mortality prediction model included LOH, CCI, and hematocrit levels.

## Figures and Tables

**Table 1:** Demographic and clinical characteristics of patients.

Variables	VTE (+) (*n* = 33)	VTE (−) (*n* = 2060)	Overall (*n* = 2093)	*p* value
Sex				
Male	13 (39.4%)	1092 (53%)	1105 (52.8%)	0.12
Female	20 (60.6%)	968 (47%)	988 (47.2%)	
Age (years)				
≤ 65	22 (66.7%)	1345 (65.3%)	1367 (65.3%)	0.87
> 65	11 (33.3%)	715 (34.7%)	726 (34.7%)	
BMI	33.7 ± 7.1	31.1 ± 8.0	31.2 ± 8.0	0.25
Race				0.02
White	30 (90.9%)	1483 (72%)	1513 (72.3%)	
Non-White	3 (9.1%)	577 (28%)	580 (27.7%)	
Smoking	2 (6.1%)	217 (10.5%)	219 (10.5%)	0.41
History of VTE	4 (12.1%)	101 (4.9%)	105 (5%)	0.07
History of anticoagulation	6 (18.1%)	166 (8.1%)	172 (8.2%)	0.04
Charlson index	4.6 ± 2.5	3.8 ± 2.3	3.8 ± 2.3	0.04
ICU stay	11 (33.3%)	200 (9.7%)	211 (10.1%)	*p* < 0.01
Length of hospitalization (days)	20.5 ± 7.9	5 ± 6.9	5.3 ± 7.6	*p* < 0.01
Hernia type				*p*<0.01
Anterior ventral hernia	32 (97%)	1609 (78.1%)	1641 (78.4%)	
Groin Hernia	1 (3%)	451 (21.9%)	452 (21.6%)	
Postoperative anticoagulation	23 (69.7%)	1753 (85.1%)	1776 (84.9%)	0.01
Interval from admission to surgery (days)	4.6 ± 12.7	0.6 ± 2.4	0.6 ± 2.9	*p* < 0.01
Procedure				
Laparoscopic operation	0	229 (11.1%)	229 (10.9%)	0.04
Intestinal resection	10 (%)	125 (6.1%)	135 (6.5%)	*p* < 0.01
Mesh application	17 (51.5%)	1488 (72.2%)	1505 (71.9%)	*p* < 0.01
Postoperative complication				
Bleeding	0	14 (0.7%)	14 (0.7%)	0.64
Infection	5 (15.2%)	61 (3%)	66 (3.2%)	*p* < 0.01
Fistula	4 (12.1%)	16 (0.8%)	20 (0.9%)	*p* < 0.01
Abnormal reaction caused by implant	3 (9.1%)	67 (3.3%)	70 (3.3%)	0.06
Laboratory examination				
Hematocrit (%)	33.9 ± 5.1	35.9 ± 5.2	35.9 ± 5.2	0.04
Platelet (10^9^/L)	244.8 ± 162.7	241.8 ± 102	241.9 ± 103.6	0.88
WBC (10^9^/L)	9.2 ± 4.6	10.2 ± 4.5	10.2 ± 4.5	0.21
Anion gap (mmol/L)	13.1 ± 2.5	13.7 ± 3.0	13.6 ± 3.0	0.29
Bicarbonate (mmol/L)	24.6 ± 3.4	25.7 ± 3.6	25.7 ± 3.6	0.08
BUN (g/dL)	16.7 ± 8.5	19.1 ± 13.6	19.1 ± 13.6	0.32
Calcium (mg/dL)	8.3 ± 0.6	8.5 ± 0.6	8.5 ± 0.6	0.13
Chloride (mmol/L)	104.5 ± 4.1	102.9 ± 4.3	102.9 ± 4.3	0.04
Creatinine (mg/dL)	0.96 ± 0.38	1.18 ± 1.27	1.18 ± 1.26	0.34
Glucose (mg/dL)	130 ± 38.1	128 ± 47.6	128 ± 47.5	0.81
Sodium (mmol/L)	138 ± 3.8	138 ± 3.4	138 ± 3.4	0.93
Potassium (mmol/L)	4.1 ± 0.5	4.2 ± 0.6	4.2 ± 0.6	0.61

Abbreviations: BMI, Body Mass Index; BUN, blood urine nitrogen; ICU, intensive care unit; VTE, venous thromboembolism; WBC, white blood cell.

**Table 2:** Univariate and multivariate logistic regression analysis of risk factors for in-hospital VTE after emergent ventral hernia repair.

Variables	Univariate analysis		Multivariate analysis	
	OR (95% CI)	*p* value	OR (95% CI)	*p* value
Sex	0.576 (0.285–1.164)	0.13		
Age (years)	0.941 (0.454–1.951)	0.87		
BMI	1.039 (0.986–1.095)	0.16		
Race (White)	3.891 (1.183–12.798)	0.03	3.642 (0.954–13.908)	0.06
Smoking	0.548 (0.130–2.305)	0.41		
History of VTE	2.675 (0.923–7.756)	0.07	0.378 (0.063–2.277)	0.29
History of anticoagulation	0.384 (0.156–0.944)	0.04	2.800 (0.903–8.686)	0.08
Charlson index	1.147 (1.005–1.309)	0.04	1.045 (0.891–1.225)	0.56
Length of hospitalization (days)	1.067 (1.047–1.087)	*p* < 0.01	1.047 (1.021–1.074)	*p* < 0.01
Hernia type	8.970 (1.222–65.820)	0.03	0.207 (0.027–1.600)	0.13
Postoperative anticoagulation	0.403 (0.190–0.855)	0.02	0.243 (0.093–0.634)	*p* < 0.01
Interval from admission to surgery (days)	1.099 (1.050–1.150)	*p* < 0.01	1.029 (0.973–1.087)	0.32
Procedure				
Intestinal resection	6.730 (3.135–14.451)	*p* < 0.01	3.012 (1.186–7.648)	0.02
Mesh application	0.408 (0.205–0.814)	0.01	0.894 (0.400–1.995)	0.78
Postoperative complication				
Infection	5.852 (2.185–15.671)	*p* < 0.01	1.899 (0.545–6.620)	0.31
Fistula	17.621 (5.550–55.947)	*p* < 0.01	5.031 (1.067–23.734)	0.04
Abnormal reaction caused by implant	2.975 (0.886–9.991)	0.08	0.231 (0.031–1.711)	0.15
Laboratory examination				
Hematocrit (%)	0.931 (0.870–0.997)	0.04	0.969 (0.898–1.046)	0.42
Platelet (10^9^/L)	1.000 (0.997–1.004)	0.88		
WBC (10^9^/L)	0.942 (0.860–1.033)	0.21		
Anion gap (mmol/L)	0.933 (0.820–1.061)	0.29		
Bicarbonate (mmol/L)	0.923 (0.842–1.010)	0.08	1.033 (0.919–1.162)	058
BUN (g/dL)	0.982 (0.948–1.018)	0.32		
Calcium (mg/dL)	0.687 (0.433–1.090)	0.11		
Chloride (mmol/L)	1.090 (1.005–1.182)	0.04	1.076 (0.974–1.188)	0.15
Creatinine (mg/dL)	0.694 (0.343–1.404)	0.31		
Glucose (mg/dL)	1.001 (0.994–1.008)	0.81		
Sodium (mmol/L)	0.995 (0.896–1.106)	0.93		
potassium (mmol/L)	0.853 (0.466–1.560)	0.61		

Abbreviations: BMI, Body Mass Index; BUN, blood urine nitrogen; ICU, intensive care unit; VTE, venous thromboembolism; WBC, white blood cell.

**Table 3:** Causes of in-hospital and 1-year mortality.

Causes of mortality	In-hospital mortality (*n* = 17)	1-year mortality (*n* = 86)
Acute mesenteric ischemia	10	56
Perforation of intestine	7	30
Postoperative complications		
Intestinal adhesions	1	26
Intra-abdominal infection	1	35
Hemorrhage	1	14
Septic shock	10	40
Cerebral infarction	1	6
Acute kidney failure	3	16
Acute respiratory failure	2	10
Acute heart failure	1	14

**Table 4:** Univariate and multivariate logistic regression analysis of risk factors for in-hospital mortality after emergent ventral hernia repair.

Variables	Univariate analysis		Multivariate analysis	
	OR (95% CI)	*p* value	OR (95% CI)	*p* value
Sex	1.646 (0.606–4.466)	0.33		
Age (years)	3.490 (1.285–9.475)	0.01	1.056 (0.997–1.119)	0.06
BMI	1.066 (1.003–1.133)	0.04	1.066 (0.969–1.173)	0.19
Race (White)	0.545 (0.206–1.438)	0.22		
Smoking	2.663 (0.861–8.241)	0.09	5.324 (1.260–22.502)	0.02
History of VTE	0.852 (0.112–6.489)	0.88		
History of anticoagulation	1.534 (0.348–6.766)	0.57		
In-hospital VTE	0.266 (0.034–2.062)	0.21		
Charlson index	1.659 (1.422–1.935)	*p* < 0.01	1.646 (1.308–2.072)	*p* < 0.01
Length of hospitalization (days)	1.055 (1.034–1.077)	*p*<0.01	1.041 (0.999–1.084)	0.06
Hernia type	2.076 (0.473–9.110)	0.33		
Postoperative anticoagulation	2.873 (0.380–21.739)	0.31		
Interval from admission to surgery (days)	1.076 (1.028–1.127)	*p* < 0.01	1.035 (0.949–1.130)	0.43
Procedure				
Intestinal resection	17.411 (6.605–45.895)	*p* < 0.01	11.597 (3.288–40.909)	*p* < 0.01
Mesh application	0.436 (0.167–1.136)	0.09	1.732 (0.511–5.877)	0.38
Postoperative complication				
Bleeding	9.918 (1.224–80.396)	0.03	10.669 (0.500–22.957)	0.13
Abnormal reaction caused by implant	3.937 (0.883–17.559)	0.07	0.420 (0.046–3.811)	0.44
Laboratory examination				
Hematocrit (%)	0.858 (0.782–0.941)	*p*<0.01	0.948 (0.852–1.056)	0.33
Platelet (10^9^/L)	0.999 (0.994–1.004)	0.65		
WBC (10^9^/L)	0.924 (0.815–1.048)	0.22		
Anion gap (mmol/L)	1.132 (0.996–1.288)	0.06	1.010 (0.825–1.235)	0.93
Bicarbonate (mmol/L)	0.833 (0.748–0.928)	*p* < 0.01	0.899 (0.734–1.103)	0.31
BUN (g/dL)	1.029 (1.009–1.049)	*p* < 0.01	0.987 (0.951–1.026)	0.51
Calcium (mg/dL)	0.621 (0.350–1.103)	0.10		
Chloride (mmol/L)	1.137 (1.024–1.262)	0.02	1.064 (0.924–1.224)	0.39
Creatinine (mg/dL)	1.140 (0.942–1.379)	0.18		
Glucose (mg/dL)	1.004 (0.998–1.010)	0.15		
Sodium (mmol/L)	1.106 (0.956–1.280)	0.17		
Potassium (mmol/L)	0.642 (0.268–1.540)	0.32		

Abbreviations: BMI, Body Mass Index; BUN, blood urine nitrogen; ICU, intensive care unit; VTE, venous thromboembolism; WBC, white blood cell.

**Table 5:** Univariate and multivariate Cox regression analysis of risk factors for 1-year mortality after emergent ventral hernia repair.

Variables	Univariate analysis		Multivariate analysis	
	HR (95% CI)	*p* value	HR (95% CI)	*p* value
Sex	1.583 (1.057–2.371)	0.03	1.347 (0.736–2.466)	0.33
Age (years)	2.352 (1.592–3.474)	*p* < 0.01	1.201 (0.665–2.169)	0.54
BMI	1.018 (0.982–1.056)	0.33		
Race (White)	1.010 (0.654–1.559)	0.97		
Smoking	2.275 (1.408–3.677)	*p* < 0.01	1.892 (0.943–3.794)	0.07
History of VTE	0.764 (0.281–2.075)	0.60		
History of anticoagulation	1.342 (0.673–2.677)	0.40		
In-hospital VTE	0.670 (0.165–2.724)	0.576		
Charlson index	7.606 (5.155–11.222)	*p* < 0.01	1.325 (1.199–1.464)	*p* < 0.01
Length of hospitalization (days)	1.039 (1.030–1.048)	*p* < 0.01	1.027 (1.002–1.053)	0.04
Hernia type	0.658 (0.430–1.007)	0.05	0.560 (0.281–1.113)	0.10
Postoperative anticoagulation	1.231 (0.688–2.202)	0.49		
Interval from admission to surgery (days)	1.047 (1.024–1.071)	*p* < 0.01	0.966 (0.888–1.051)	0.42
Procedure				
Laparoscopic Operation	0.079 (0.011–0.563)	0.01		
Intestinal Resection	2.653 (1.534–4.590)	*p* < 0.01	0.650 (0.250–1.691)	0.38
Mesh Application	0.649 (0.435–0.970)	0.04	0.856 (0.470–1.557)	0.61
Postoperative complication				
Bleeding	1.527 (0.213–10.947)	0.67		
Infection	1.988 (0.871–4.536)	0.10		
Fistula	3.245 (1.029–10.233)	0.04	2.085 (0.392–11.094)	0.39
Abnormal reaction caused by implant	1.515 (0.617–3.722)	0.37		
Laboratory examination				
Hematocrit (%)	0.907 (0.871–0.945)	*p* < 0.01	0.927 (0.877–0.980)	*p* < 0.01
Platelet (10^9^/L)	0.998 (0.996–1.000)	0.07	0.999 (0.996–1.002)	0.54
WBC (10^9^/L)	0.984 (0.937–1.035)	0.54		
Anion gap (mmol/L)	1.124 (1.061–1.192)	*p* < 0.01	1.084 (0.969–1.212)	0.16
Bicarbonate (mmol/L)	0.900 (0.854–0.948)	*p* < 0.01	1.058 (0.974–1.150)	0.18
BUN (g/dL)	1.030 (1.021–1.039)	*p* < 0.01	1.004 (0.987–1.022)	0.61
Calcium (mg/dL)	0.649 (0.481–0.875)	*p* < 0.01	0.934 (0.584–1.493)	0.78
Chloride (mmol/L)	1.012 (0.963–1.064)	0.63		
Creatinine (mg/dL)	1.158 (1.080–1.242)	*p* < 0.01	1.037 (0.880–1.221)	0.67
Glucose (mg/dL)	0.999 (0.994–1.004)	0.76		
Sodium (mmol/L)	1.003 (0.941–1.069)	0.93		
Potassium (mmol/L)	0.833 (0.579–1.196)	0.32		

Abbreviations: BMI, Body Mass Index; BUN, blood urine nitrogen; ICU, intensive care unit; VTE, venous thromboembolism; WBC, white blood cell.

## Data Availability

The datasets generated during and/or analyzed during the current study are available from the corresponding author upon reasonable request.

## Nomenclature

VTE: Venous thromboembolism
EVHR: Emergent ventral hernia repair
DVT: Deep vein thrombosis
PE: Pulmonary embolism
SVT: Splanchnic vein thrombosis
EGS: Emergency general surgery
ICU: Intensive care unit
CCI: Charlson Comorbidity Index
LOH: Length of hospitalization
WBC: White blood cell
HCT: Hematocrit
PLT: Platelet count
ORs: Odds ratios
HR: Hazard ratio
CIs: Confidence intervals
ROC: Receiver operating characteristic curve
AUC: Area under the ROC curve
DCA: Decision curve analysis

## Supporting Information

Graphical abstract.
