# Supplementary figures and images for: Risk Factors of In-Hospital Venous Thromboembolism and Prognosis After Emergent Ventral Hernia Repair
Source: Emerg Med Int. 2024 Nov 12;2024:6670898. doi: 10.1155/2024/6670898 (PMC11576084; doi:10.1155/2024/6670898)

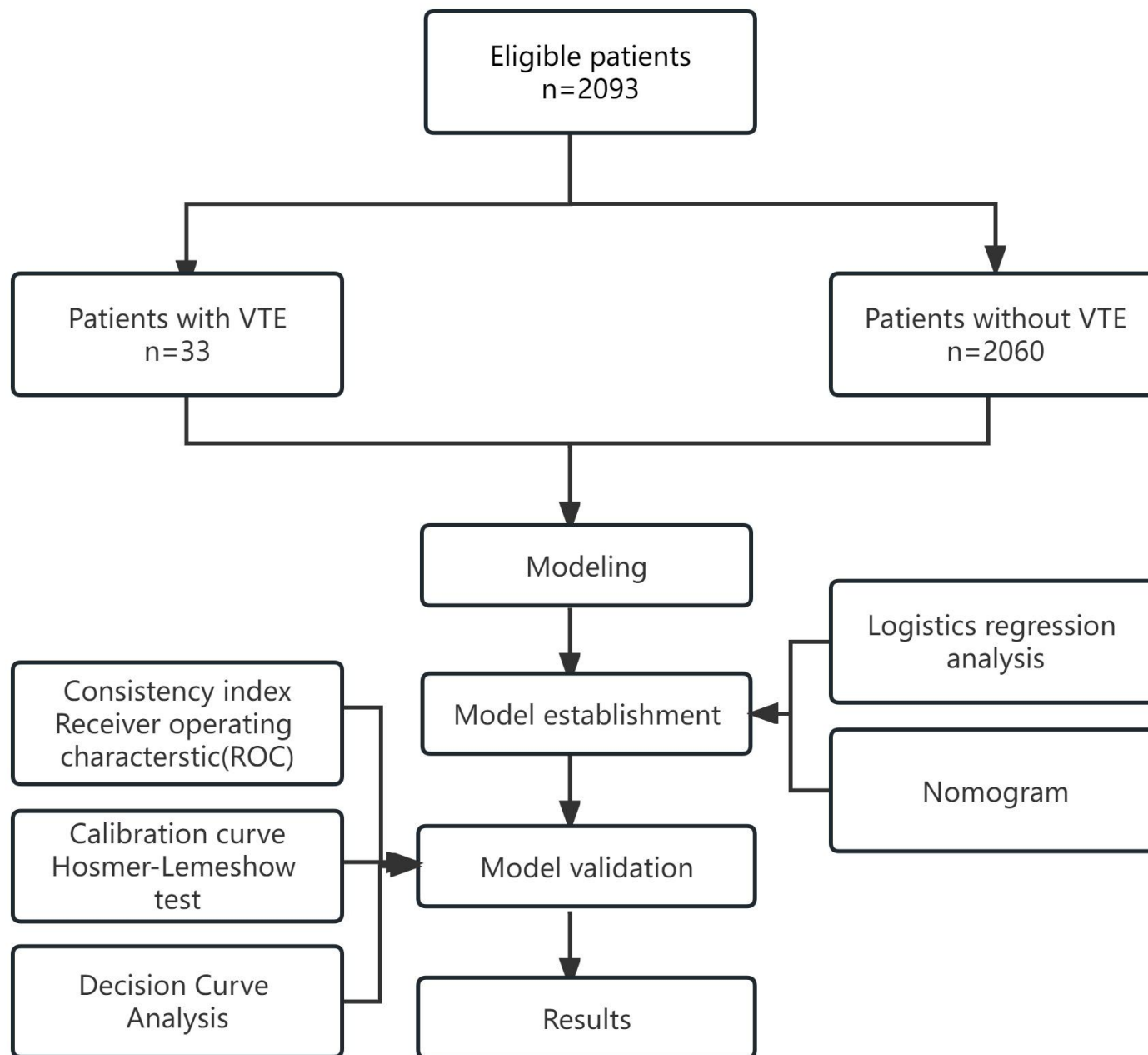

Supplement: Supporting Information — Additional supporting information can be found online in the Supporting Information section. [file 6670898.f1.pdf]
